# Response Criteria in Advanced Systemic Mastocytosis: Evolution in the Era of KIT Inhibitors

**DOI:** 10.3390/ijms22062983

**Published:** 2021-03-15

**Authors:** William Shomali, Jason Gotlib

**Affiliations:** Division of Hematology, School of Medicine, Stanford Cancer Institute/Stanford University, 875 Blake Wilbur Drive, Stanford, CA 94305-6555, USA; wshomali@stanford.edu

**Keywords:** systemic mastocytosis, *KIT* D816V, midostaurin, avapritinib, IWG-MRT-ECNM

## Abstract

Systemic mastocytosis (SM) is a rare clonal hematologic neoplasm, driven, in almost all cases, by the activating *KIT* D816V mutation that leads to the growth and accumulation of neoplastic mast cells. While patients with advanced forms of SM have a poor prognosis, the introduction of KIT inhibitors (e.g., midostaurin, and avapritinib) has changed their outlook. Because of the heterogenous nature of advanced SM (advSM), successive iterations of response criteria have tried to capture different dimensions of the disease, including measures of mast cell burden (percentage of bone marrow mast cells and serum tryptase level), and mast cell-related organ damage (referred to as C findings). Historically, response criteria have been anchored to reversion of one or more organ damage finding(s) as a minimal criterion for response. This is a central principle of the Valent criteria, Mayo criteria, and International Working Group-Myeloproliferative Neoplasms Research and Treatment and European Competence Network on Mastocytosis (IWG-MRT-ECNM) consensus criteria. Irrespective of the response criteria, an ever-present challenge is how to apply response criteria in patients with SM and an associated hematologic neoplasm, where the presence of both diseases complicates assignment of organ damage and adjudication of response. In the context of trials with the selective *KIT* D816V inhibitor avapritinib, pure pathologic response (PPR) criteria, which rely solely on measures of mast cell burden and exclude consideration of organ damage findings, are being explored as more robust surrogate of overall survival. In addition, the finding that avapritinib can elicit complete molecular responses of *KIT* D816V allele burden, establishes a new benchmark for advSM and motivates the inclusion of definitions for molecular response in future criteria. Herein, we also outline how the concept of PPR can inform a proposal for new response criteria which use a tiered evaluation of pathologic, molecular, and clinical responses.

## 1. Introduction

Systemic mastocytosis (SM) is a clonal, multisystem disease, driven by activating *KIT* mutations, most commonly D816V, that leads to accumulation of neoplastic mast cells in various organs [1]. This abnormal growth of mast cells results in organ damage in addition to mast cell-related mediator symptoms. Advanced SM (advSM) is an umbrella term encompassing the variants aggressive SM (ASM), SM with an associated hematologic neoplasm (SM-AHN), and mast cell leukemia (MCL), which are associated with a poor prognosis and decreased survival [2,3]. The introduction of KIT inhibitors (e.g., midostaurin, and avapritinib) has resulted in a paradigm shift in the treatment of advSM and has prompted a re-evaluation how to optimize response criteria in order to best capture clinical benefit and long-term outcomes such as overall survival. In addition to measuring meaningful health outcomes, endpoints should be quantifiable, reproducible, and practical [4]. One of the “practical” purposes of response criteria is to harmonize response evaluation across trials so that the efficacy of novel therapeutics can be suitably compared. At the same time, however, they should be user-friendly and readily applicable in community practice. 

## 2. Response Criteria in AdvSM

### 2.1. Valent Criteria

AdvSM encompasses complex variants necessitating expertise in the diagnosis and response evaluation of different therapies. The first treatment response criteria, referred to as Valent criteria, were proposed in 2003 and reaffirmed in 2007 (Table 1) [5,6]. They were based on the diagnostic and classification criteria published earlier in 2001 that defined organ damage (C findings) in patients with ASM [7]. C findings include cytopenias (ANC < 1 × 10^9^/L, hemoglobin <10 g/dL, platelet count <100 × 10^9^/L); hepatomegaly with ascites, abnormal liver function and/or portal hypertension; splenomegaly with hypersplenism; malabsorption leading to hypoalbuminemia and weight loss; and/or bone lesions with large osteolyses or severe osteoporosis with pathologic fractures [7]. Valent criteria were based on reversal of C findings and divided into major response (MR), partial response (PR) and no response [5]. Patients were deemed to have MR if they had complete resolution of at least one C finding without progression of other C findings. MR is divided into three subcategories: (A) complete remission (CR) (resolution of abnormal MC infiltrates in organs, decrease of serum tryptase levels to less than 20 ng/mL, and disappearance of SM-associated organomegaly), (B) incomplete remission (decrease of MC infiltrates and/or serum tryptase levels, and/or visible regression of organomegaly (ad hoc defined as >50% reduction without complete normalization), and (C) pure clinical response (without decrease of MC infiltrates, serum tryptase levels, or organomegaly). A partial response (PR) is defined as incomplete regression of 1 or more C findings (good partial response, ≥50% regression of ≥1 C findings; and minor response, <50% regression). Patients with stable or any progressive C finding (ad hoc defined as 50% worsening of one or more C findings from baseline) are categorized as non-responders (Table 1). These criteria were initially applied to 14 patients with ASM who were previously treated with interferon-alfa and showed a MR of 21.4%, PR in 35.7%, and no response in 42.9% of patients receiving this therapy, highlighting the limited efficacy of interferon-alfa in advSM [5]. In addition to trials of interferon-alfa, Valent criteria were used to adjudicate responses in patients with advSM treated with cladribine [8], as well as the tyrosine kinase inhibitors (TKIs) imatinib, dasatinib, and nilotinib [9,10,11].

Valent response criteria were an important step to help adjudicate responses in clinical trials but had some limitations pertaining to the difficulty in quantifying some C findings (e.g., ascites, and bone involvement), and without a defined minimum change in a C finding in case of response or progression. For example, the C-finding of hypoalbuminemia could be considered “normalized” if a value just below normal range (e.g., 3.4 mg/dL) increased to 3.6 mg/dL, within the reference range of 3.5–5.0 mg/dL for serum albumin. Such a small change in albumin, although reverting to normal, would not be considered clinically significant. In addition, duration of response (e.g., ≥8 weeks) and the criteria for red blood cell and platelet transfusion dependence were not defined in these criteria and instead were adopted from the IWG criteria for myelodysplastic syndromes (MDS) [12] and applied to the phase 2 investigator-initiated [13] and industry-sponsored [14] studies of midostaurin. These changes were referred to as “modified Valent response criteria” and “Cheson criteria for transfusions” and together were used to calculate overall response rate (ORR) in these trials.

### 2.2. Mayo Criteria

Based on some of the aforementioned limitations of the Valent criteria, the Mayo Clinic group proposed revised criteria for advSM in 2010 that were based on (A) disease related symptoms, (B) organomegaly/lymphadenopathy, (C) organ damage (referred to as disease-related organopathy), and (D) bone marrow findings (Table 2) [4]. These criteria used the National Cancer Institute’s Common Terminology Criteria for Adverse Events (CTCAE) v3.0 in defining organ damage and importantly, minimal required improvement from baseline to be considered a response (or progression). As Table 2 highlights, complete response, major response, partial response, stable disease, and progressive disease are based on one or more of these four criteria being met. These criteria required a minimal response duration of four weeks. SM with an associated hematologic neoplasm (SM-AHN) and MCL were not included in these new set of criteria and response evaluation specific to the AHN component and acute leukemia were suggested for those subtypes, respectively. 

### 2.3. International Working Group-Myeloproliferative Neoplasms Research and Treatment & European Competence Network on Mastocytosis (IWG-MRT-ECNM) Response Criteria

The International Working Group-Myeloproliferative Neoplasms Research and Treatment and European Competence Network on Mastocytosis consensus response criteria (henceforth referred to as IWG) were developed to build upon and overcome the limitations of prior response criteria [2]. Extending the Mayo concept, the IWG criteria include comprehensive definitions of organ damage eligible for response evaluation using CTCAE grading, where organ dysfunction is required to be ≥grade 2, and specific criteria for clinical improvement are defined (Table 3). The definitions of “eligible organ damage” are distinct from the WHO definitions of “C findings”. For example, (large) osteolytic bone lesions and/or pathologic fractures and weight loss are WHO-defining C findings but not eligible for response assessment in IWG criteria given the challenge in their quantification. For instance, it is often difficult to obtain medical documentation of weight loss; weight loss may be relatively distant in the patient’s medical history and stable just before drug start; and changes in weight may be confounded by shifts in ascites or peripheral edema. In patients with baseline small-volume pleural effusions (not included in prior response criteria) and/or ascites, it may be challenging to characterize their resolution as clinically meaningful; therefore, in the context of the IWG, such organ damage is only considered eligible if diuretics and/or use of repeated thoracentesis or paracentesis are required for their management in the 12 weeks prior to study entry. Symptomatic splenomegaly (>5 cm below costal margin) is also included as a form of eligible organ damage, which is different from the WHO C finding of “splenomegaly with hypersplenism”. 

IWG responses are anchored to normalization of one or more eligible organ damage findings which forms the basis of clinical improvement (CI). A PR additionally requires ≥50% reduction in the serum tryptase level and in the percentage of neoplastic mast cells in the BM and/or another extracutaneous organ. A CR requires normalization of all baseline IWG organ damage findings, reduction of the serum tryptase level to <20 ng/mL, elimination of mast cell aggregates in the BM (or other extracutaneous organ), peripheral blood count recovery to an absolute neutrophil count >1 × 10^9^/L, Hb ≥ 11 g/dL and platelet count ≥100 × 10^9^/L, and complete resolution of palpable hepatosplenomegaly and biopsy-proven or suspected SM-related organ damage. Criteria for progressive disease (PD) and loss of response (LOR) are also detailed in the IWG criteria, and stable disease is defined as not meeting criteria for CR, PR, CI, or PD. Responses (CR, PR, CI, or SD) must be confirmed for ≥12 weeks, whereas LOR and PD must persist for ≥8 weeks to be confirmed. Those criteria were adopted by regulatory agencies (The United States Food and Drug Administration (FDA) and European Medicines Agency (EMA)) for clinical trial response evaluation. For example, the phase II trial of brentuximab vedotin in patients with CD30^+^ advSM was the first study to apply these published criteria [15].

## 3. Tyrosine Kinase Inhibitors in AdvSM and Challenges for Response Criteria

### 3.1. Imatinib

Currently, imatinib and midostaurin are the two KIT inhibitors that are FDA-approved for the treatment of ASM and advSM, respectively. Imatinib was FDA-approved in 2006 for adult patients with ASM without the *KIT* D816V mutation or with unknown *KIT* mutational status. The approval was based on 28 patients; 5 patients were enrolled in a prospective clinical trial [16], and 23 patients were reported in 10 case reports and case series [17]. Eight patients had complete hematologic response to imatinib; of whom seven patients had a *FIP1L1-PDGFRA* fusion gene detected (an entity which mimics SM with elevated serum tryptase and increased bone marrow mast cells), and one patient had chronic myeloid leukemia with concurrent ASM (*KIT* D816V mutated). Two patients with *KIT* juxtamembrane mutations (F522C and K509I) had a partial hematologic response, similar to the 15 patients with unknown *KIT* mutational status. Three patients with *KIT* D816V had no response to imatinib [17]. Response criteria used in evaluating the activity of imatinib in those 23 patients were variable, generally by evaluating normalization of blood counts and bone marrow morphology. Additional data highlight successful use of imatinib in patients with the well-differentiated SM variant, known to harbor non-exon 17 mutations [18]. 

### 3.2. Midostaurin

Midostaurin, a multikinase inhibitor active against *KIT* D816V mutation, was approved by the FDA and EMA in 2017 for the treatment of patients with advSM after undergoing evaluation in 2 open label phase II studies [13,14]. In the first investigator-initiated study of 26 patients, 100 mg twice daily of midostaurin led to an ORR and MR of 69% and 50%, respectively, based on modified Valent criteria and Cheson criteria for transfusions, with two patients achieving CR on long-term follow up [13]. The median overall survival (OS) in this trial was 40 months. Those encouraging results were confirmed in a global registration trial that included 116 patients, 89 of whom were evaluable with ≥1 C finding and showed an ORR and MR rate of 60% and 45%, respectively [14]. Median OS was 28.7 months and progression-free survival was 14.1 months. Although, no randomized comparison has been done to date, midostaurin treatment was associated with improved OS compared to historical controls [19,20]. The most frequent adverse events (AEs) were low-grade nausea, vomiting, and diarrhea. New or worsening grade 3 or 4 neutropenia, anemia, and thrombocytopenia occurred in 24%, 41%, and 29% of patients, respectively, mostly in those with preexisting cytopenias [14].

The FDA and EMA conducted post-hoc exploratory analyses of the global midostaurin trial by IWG criteria using an algorithmic approach. The FDA included 115 response evaluable patients and cited an ORR of 17% consisting of 2% CR and 15% PR [21]. In contrast to the FDA, the EMA included the response category of CI in their ORR, which was 28%, consisting of 1% CR, 15% PR, and 12% CI among 113 evaluable patients [22]. Regulatory authority concerns about how to interpret responses are discussed below, and have informed new iterations of response criteria, including pure pathologic response (PPR), which is currently under development.

### 3.3. Avapritinib

Avapritinib (BLU-285) is a potent, selective inhibitor of *KIT* D816V, with pre-clinical studies demonstrating 10-fold more potency against this mutant kinase compared to midostaurin [23]. Avapritinib also exerts activity against platelet-derived growth factor receptor alfa (*PDGFRA*) mutants, and was approved by the FDA in 2020 for the treatment of adults with unresectable or metastatic gastrointestinal stromal tumor (GIST) harboring *PDGFRA* exon 18 mutations [24,25]. In the context of advanced SM, avapritinib has undergone phase I evaluation (EXPLORER; NCT02561988) with encouraging results [26,27], and a phase II study (PATHFINDER; NCT03580655) has recently completed accrual. 

Worsening cytopenias observed with midostaurin (and avapritinib as discussed below) highlights the myelosuppressive effect of KIT inhibition and led to the modification of IWG criteria for the avapritinib clinical trials (modified IWG (mIWG)), to include a CR with partial hematologic recovery (CRh) category, which would otherwise have been considered a PR by the published IWG criteria. CRh requires all criteria for CR but allows for residual cytopenias defined by ≥1 of the following: absolute neutrophil count ≥0.5 × 10^9^/L, Hb ≥ 8 g/dL, and/or platelet count ≥50 × 10^9^/L that are considered unrelated to SM (e.g., related to drug effect, AHN component or other comorbidities). 

In addition to the modification of IWG criteria to include CRh as discussed above, the response criteria were further modified to allow for documented weight loss of ≥10% over the previous 24 weeks (+/− 12 weeks) as an eligible organ damage finding, despite the aforementioned caveats of interpreting changes in weight. Furthermore, the mIWG criteria allow patients with palpable splenomegaly ≥5 cm (compared to >5 cm in the published criteria) to be enrolled, irrespective of splenomegaly-related symptoms, given the subjectivity in attributing abdominal symptoms to splenomegaly. This does raise, however, the issue of enrolling individuals with the “sole” finding of splenomegaly as an “eligible” mIWG organ damage into trials of advSM, since this is a B finding which is more consistent with a diagnosis of smoldering SM (SSM) whose diagnosis requires ≥2 B findings. Lastly, mIWG criteria also allow for PD confirmation after four weeks instead of eight weeks since patients might need to start new therapy prior to the eight-week confirmation window in order to address worsening disease. Major differences between the original and modified IWG criteria are summarized in Table 3.

The results of the phase I dose escalation and expansion phases have been recently updated and showed an ORR of 75% in 53 evaluable patients, based on mIWG criteria, with 36% of patients achieving either a CR or CRh, 34% PR, 6% CI, and 23% SD. Rates of ORR/CR+CRh among midostaurin-naïve (*n* = 36) and previously treated midostaurin patients (*n* = 17) were 83/44% and 59/18%, respectively. The median overall survival was 46.9 months for all advSM patients (as well as the SM-AHN subgroup) and was not reached for ASM and MCL patients. The estimated 24-month OS was 75% (100% in ASM, 92% in MCL and 68% in SM-AHN) [26]. 

Ninety-nine percent of patients with marrow *KIT* D816V evaluation at baseline achieved ≥50% reduction in *KIT* D816V allele burden using digital droplet PCR, with *KIT* allele fraction decreasing to <1% in 68% of patients [27]. Molecular responses in *KIT* D816V allele burden (using expressed allele burden by RNA) are associated with response and prolonged OS in a post-hoc analysis of 38 advSM patients treated with midostaurin, and may similarly prove to be prognostic with avapritinib [28]. Although not formally currently included in the IWG criteria or its modification, assessing molecular responses as a measure of depth of response “measurable residual disease status” is a response benchmark that should now be incorporated into newly devised response criteria, and reflects the potential disease-modifying activity of selective *KIT* D816V inhibition. 

## 4. Challenges of Response Adjudication and Concerns of the Regulatory Authorities

Response criteria in SM combine complex pathologic, laboratory, and clinical assessments to adjudicate responses, and these determinations can be confounded by the AHN component, drug side effects, and/or other comorbidities. With submission of the midostaurin data (which is based on modified Valent criteria and Cheson criteria for transfusions) to the regulatory agencies, questions arose regarding how to interpret levels of response and their associated clinical benefit. The regulatory agencies highlighted the heterogeneity in the degree of response within response categories, subjectivity in response assessment, and varying stringency in the criteria to define organ damage responses. A significant concern was that a large majority of patients not only suffered from SM, but also from an associated hematologic neoplasm. In turn, this created uncertainty about whether splenomegaly and lab abnormalities were caused by the SM or the AHN component. In order to mitigate these concerns, the FDA conducted the aforementioned post hoc analysis with the IWG criteria using an algorithmic approach and their reviewers recommended that only CR and PR be included in the ORR for the purposes of the midostaurin application [21]. The EMA also performed a post hoc analysis using IWG criteria, but included CI which assumes some recognition of the clinical value of improving organ damage alone without the requirement for reduction in measures of mast cell burden such as BM mast cell aggregates or the serum tryptase level which are required in order to achieve a PR or CR [22].

## 5. Pure Pathologic Response (PPR) Criteria

While published IWG criteria and its modified version provide more stringent and clinically relevant definitions of organ damage eligibility and response, defining these responses is complex due to their heterogenous nature. The response criteria are geared more for clinical trials and are challenging to implement in clinical practice. In some patients, a discordance may exist between lingering non-hematologic organ damage findings, such as an improving, but persistently elevated alkaline phosphatase level, with simultaneous complete elimination of BM mast cell aggregates and reduction of the serum tryptase level to < 20 ng/mL. This scenario is not uncommon, and results in the downgrade of a response from CR to SD if no other organ damage findings are present or if other organ damage findings have not normalized.

Trials in advanced SM have *pro forma* required one or more organ damage/C finding(s) to be present in order for patients to be eligible for enrollment. This is because response criteria are anchored to improvement of organ damage as a minimal requirement for all response categories. However, advSM patients that have been previously treated with other therapies such as midostaurin may no longer exhibit organ damage findings or they are not clearly related to SM, but to recent therapy. Such individuals may be excluded from trial participation despite having measurable mast cell disease (increased bone marrow mast cell burden and/or tryptase level), thus limiting the evaluable population of advSM patients. Indeed, focusing on pathologic and molecular responses may be more strongly associated with clinical outcomes such as survival, and therefore may be more favored by regulatory agencies. To date, the depth of responses (i.e., CR vs. PR vs. CI vs. SD) has not been shown to correlate with overall survival.

In order to overcome the potential challenges and obstacles posed by complex C-finding assessments, pure pathologic response (PPR) criteria were recently proposed [26] (Table 4). PPR criteria include CR/CRh which requires resolution of bone marrow mast cell aggregates, a serum tryptase < 20 ng/mL, and either a full (CR) of partial (CRh) hematologic recovery; PR which is defined as ≥50% reduction in bone marrow mast cells and serum tryptase level; progressive disease (PD), which is defined as transformation to acute myeloid leukemia (AML) or mast cell leukemia (MCL); and stable disease (SD), which does not meet criteria for CR/CRh, PR, or PD. These criteria are restricted to changes in bone marrow mast cell burden and serum tryptase level, and additionally assess the degree of molecular response by assessing reduction in *KIT* D816V mutant allele burden using a sensitive assay with a limit of detection in the range of ~0.1%.

PPR criteria were evaluated in 53 patients who were response-evaluable by mIWG criteria in the phase I avapritinib trial [26]. PPR yielded a similar ORR, but a higher CR/CRh rate (47% vs. 36%), demonstrating the discordance between pathologic and clinical responses. In addition, a complete molecular response was observed in 25% of the patients. Using PPR criteria also increases the evaluable patient population. This is particularly relevant to patients without baseline IWG findings which may relate to recent prior therapy, such as cladribine or midostaurin, and/or the use corticosteroids which are often used as a temporizing measure to mitigate rebound symptoms but may also erase organ damage findings after washout of prior therapy. Applying these PPR criteria in the phase I avapritinib trial allowed 11 additional advSM patients lacking mIWG findings to be evaluable.

In a landmark analysis starting at the end of cycle 6, patients who were deemed as responders (CR/CRh/PR) by PPR criteria had two-year OS of 86% whereas non-responders (all SD) had two-year OS of 58% (*p* = 0.013) [26]. This statistically significant difference was retained when comparing the different levels of response (e.g., CR/CRh: 100% vs. PR: 81% vs. SD: 58%) (*p* = 0.026). In comparison, responders by mIWG criteria (CR/CRh/PR/CI) had two-year OS of 85% (CR/CRh, 100%; PR, 81%; CI, 86%), whereas non-responders had two-year OS of 59% (*p* = 0.083), consistent with a trend in improvement in OS. However, statistical significance was not reached when comparing individual response categories and their differential impact on overall survival (*p* = 0.253 when comparing CR/CRh vs. PR vs. CI vs. SD) [26].

## 6. Proposed Response Criteria to Meet the Era of KIT Inhibitors

**Figure 1:**
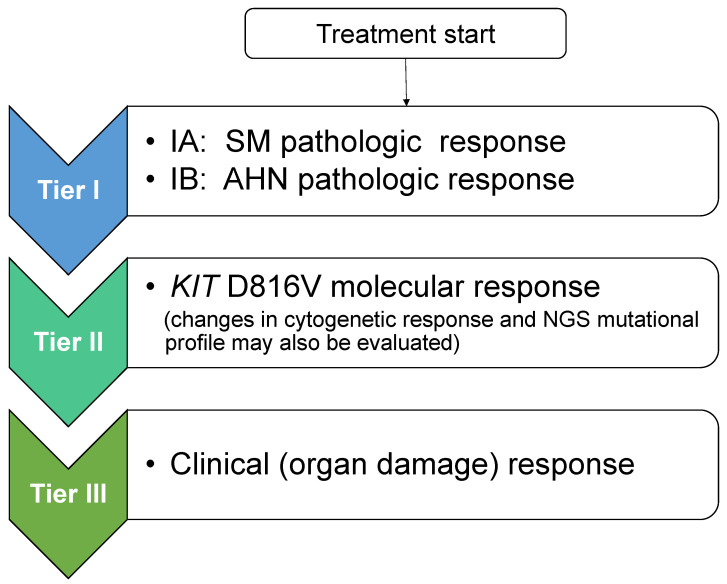
Proposal for Tiered Response Criteria in Advanced Systemic Mastocytosis. After initiation of treatment, adjudication of response is separated into three sequential tiers. Evaluation commences with tier I: SM pathologic response (and if an SM with an associated hematologic neoplasm (SM-AHN) is present, concurrent pathologic assessment of the AHN component is undertaken; tier II: *KIT* D816V molecular response (evaluation of changes in cytogenetics and next generation sequencing mutation profile may also be undertaken at this step); and tier III: evaluation of clinical (organ damage) response using IWG or modified IWG criteria. Although not shown, a tier IV level of response evaluating symptoms (e.g., using the advanced systemic mastocytosis-symptom assessment form (AdvSM-SAF)) and other patient-reported outcome instruments evaluating quality of life may be considered. NGS: next-generation sequencing.

Taking into consideration the PPR concept, new response criteria for advSM were recently proposed (Jason Gotlib and Andreas Reiter, European Competence Network on Mastocytosis 2020 Annual Meeting, Vienna, Austria) that follow a modular approach and create a tiered response evaluation of pathologic, molecular, and clinical responses (Figure 1). Tier I includes SM pathologic response (based on PPR criteria), and if present, assessment of AHN pathologic response based on published AHN-specific response criteria; tier II includes *KIT* D816V molecular response (and may include cytogenetics and results from next generation sequencing [NGS] mutation profiling); and tier III includes clinical (organ damage) response according to (m)IWG criteria. As shown in Table 5, when translating these tiered levels of response evaluation to study objectives in a hypothetical trial of a novel agent, tier I pathologic evaluation of SM would serve as the primary endpoint, and tiers II and III would be incorporated as secondary endpoints with additional trial objectives. These modules of pathologic, molecular/cytogenetic, and clinical response can be incorporated into a multivariate analysis to assess their impact on disease outcomes such as overall and progression-free survival, as well as transformation to AML. These sequential, tiered response criteria allow uncoupling of pathologic and clinical responses. For example, if clearance of bone marrow mast cell infiltrates and normalization of the serum tryptase level were achieved (which would define a CR or CR_h_ by PPR criteria), the presence of lingering organ damage findings would not downgrade the overall response. Such criteria simplify adjudication of response by relying solely on measures of mast cell burden without the need to integrate organ damage response(s) in the overall response assessment which can often be confounded by drug effects, co-morbid conditions, and/or by the AHN, if present. Conversely, it is possible that pathologic and molecular responses may lag behind clinical responses in some patients; therefore, sufficient time on therapy should be allocated to allow deeper responses to emerge.

As has been undertaken with the EXPLORER and PATHFINDER studies of avapritinib, analysis of *KIT* D816V molecular responses requires a high sensitivity assay such as allele-specific quantitative PCR or digital droplet PCR. This can facilitate assessment of measurable residual disease (MRD) and its impact on long term outcomes. Pre-trial and on-treatment NGS using multi-gene panels should also be included as a standard secondary endpoint to analyze additional somatic mutations beyond *KIT* D816V, especially high-risk mutations (*SRSF2*, *ASXL1*, and *RUNX1*, e.g., *S/A/R*) which are commonly present in SM-AHN. Since these mutations reflect multilineage disease, e.g., involvement of mast cells and other myeloid cell types [29,30,31,32], novel single cell sequencing techniques might be an interesting exploratory endpoint in this setting. This can be used to further dissect the clonal landscape of mutationally complex advSM and to better understand mechanisms of resistance under the pressure of KIT inhibition.

Patient reported outcomes (PROs) incorporating symptom scores and measures of quality of life are key in assessing clinical benefit and are increasingly being adopted by regulatory agencies during the drug approval process. This is exemplified by the use of the myeloproliferative neoplasm-symptom assessment form (MPN-SAF) and abridged 10-symptom total symptom score (TSS-10) with JAK inhibitors and other agents in myelofibrosis [33]. In the global midostaurin trial, the Memorial Symptom Assessment Scale (MSAS) was used to evaluate changes in patient-reported symptoms across 32 symptom domains, and quality of life was assessed using the Medical Outcomes Study 12-Item Short-Form Health Survey (SF-12) [14]. An advSM symptom assessment form (AdvSM-SAF) was the first validated PRO tool designed specifically to assess advSM symptoms and was incorporated into the trials of avapritinib [34]. Avapritinib elicited a 40% mean reduction in total symptom score compared to baseline, and a 60% mean reduction in the most bothersome symptom domain (GI or skin). Using the EORTC-QLQ-C30 instrument, avapritinib also demonstrated a significant improvement in QOL that approached healthy, age-matched controls. While such PROs are not included in the current IWG criteria or its modification, their inclusion in the newly proposed response criteria, e.g., as a tier 4 secondary endpoint, should be encouraged.

## 7. Conclusions and Future Challenges

KIT inhibition has transformed the treatment paradigm of advSM but has also prompted a re-evaluation of how to tailor response criteria to best capture the clinical benefit of KIT-targeting drugs. Lessons learned from midostaurin and avapritinib clinical trials have led to modifications of the IWG criteria and an increasing recognition that more in-depth responses are now feasible. Therefore, new response criteria that are anchored to histopathologic responses, as well as molecular responses are now warranted, and should be uncoupled from the potential confounding effects of incompletely resolved organ damage. PPR criteria are aligned with this imperative. They are focused solely on measures of mast cell burden, avoid the challenges of adjudicating organ damage, can be easily used in clinical practice, and can be applied to any advSM patient with a measurable mast cell burden. The tiered system of response evaluation builds upon the PPR by prioritizing pathologic response as a primary trial endpoint and other tiers of response are separately incorporated as secondary trial endpoints, providing a composite picture of additional measures of clinical benefit.

Since 60–70% of advSM patients carry a diagnosis of SM-AHN, assessment of histopathologic response of the AHN component will be critical, and has been historically ignored in trials of advSM with KIT inhibitors, with the exception of characterizing peripheral blood monocytosis and eosinophilia. In the era of KIT inhibitors, progression of the AHN component (i.e., progression to secondary AML) continues to be the driver of survival in most patients with SM-AHN. In this regard, consensus is needed to define progression (e.g., only transformation to AML, or also progression of an AHN from low- to high-risk (e.g., CMML-1 to CMML-2)). In a similar vein, detailed criteria are needed to define what constitutes an “event” leading to termination of a trial participant (e.g., disease progression, a drug-related adverse event, or other reasons for study withdrawal). Generating such criteria will help define endpoints such as event-free survival (EFS), progression-free survival (PFS), and leukemia-free survival (LFS). These issues will become increasingly relevant as combination trials using KIT inhibitors and AHN-targeting therapies are developed.

Finally, the finding that hereditary α-tryptasemia (HαT) is enriched in patients with SM adds a layer of complexity in assessing symptom scores and both baseline and on-treatment tryptase levels [35,36,37]. Screening patients enrolled in clinical trials for HαT might help further interpret changes in serum tryptase and symptom burden in the subgroup of patients with concomitant HαT [38].

## Figures and Tables

**Table 1:** Valent Response Criteria in aggressive systemic mastocytosis (ASM) and mast cell leukemia (MCL).

**Major Response**
Complete resolution of at least one (one or more) C-finding(s) and no progression in other C-findings (A) Complete remission: disappearance of mast cell infiltrates in affected organs, decrease of serum tryptase to <20 ng/mL, and disappearance of SM-associated organomegaly (B) Incomplete remission: decrease in mast cell infiltrates in affected organs, and/or substantial decrease of serum tryptase level, and/or visible regression of organomegaly (C) Pure clinical response: without decrease in mast cell infiltrates, without decrease in tryptase levels, and without regression of organomegaly
**Partial Response**
Incomplete regression of one or more C-finding(s) ^a^, without complete regression, and no progression in other C-findings (A) Good partial response: >50% regression (B) Minor response: ≤50% regression
**No Response**
C-finding(s) persistent or progressive ^b^ (A) Stable disease: C-finding-parameters show constant range (B) Progressive disease: one or more C-finding(s) show progression

^a^ With or without decrease in mast cell infiltrates, serum tryptase levels, and organomegaly; ^b^ In case of progressive C-findings and documented response in other C-finding(s), the final diagnosis is still progressive disease.

**Table 2:** Mayo Clinic revised response criteria in ASM.

Response Category	Disease-Related Symptoms ^1^	Organomegaly/ Lymphadenopathy ^2^	Disease-Related Organopathy ^3^	Bone Marrow (BM) Findings ^4^
	A	B	C	D
Complete response (CR): A+B+C+D required (when present)	Complete resolution for 3 months	Complete resolution ^2^	Complete resolution ^5^	Absence of abnormal mast cell (MC) infiltration ^7^
Major response (MR): A+B+C+D required (when present)	No progression (at a minimum)	No progression (at a minimum)	Complete resolution of at least 1 element of organopathy ^3,6^	>50% decrease in BM MC (%)
Partial response (PR): A or B or C (without progression of others)	Complete resolution for 3 months	Complete resolution ^2^	≥2 grade improvement in at least 1 element of organopathy ^6,8^	No progression (at a minimum)
Stable disease (SD)	None of the above responses			
Progressive disease (PD): B or C required	Not applicable ^9^	>50% increase from baseline ^2^	≥2 grade worsening from baseline	Not applicable

Responses are validated only if they last for no fewer than 4 weeks. Correlation between clinical response and change in MC mediator level(s)** and *KIT* D816V allele burden needs further study; recommend prospective sample collection at pre-treatment and at time of peak clinical response for comparison. ** Serum tryptase, 24 -h urine histamine, methylhistamine, and 11-β-Prostaglandin F2α. ^1^ To be considered as a parameter for response measurement, symptoms must be frequent (occurrence of at least once per month), severe enough to require treatment, despite prophylaxis (H1 and H2 histamine receptor antagonists, proton pump inhibitors, and/or oral cromolyn sodium), and accompanied by either organomegaly/lymphadenopathy or organopathy. ^2^ Organomegaly/lymphadenopathy by imaging studies required at baseline; baseline and post-treatment status must be documented by imaging studies to allow third-party confirmation of response or progression. ^3^ ≥Grade 2 ascites (not optimally controlled with medical therapy) OR ≥grade 2 weight loss OR ≥grade 2 osteoporosis (large osteolytic lesions or pathologic fracture) OR ≥grade 2 anemia (Hgb < 10 g/dL) OR thrombocytopenia (platelet count < 75 × 10^9^/L) OR ≥grade 2 hyperbilirubinemia or hypoalbuminemia that is a disease-related change from baseline (grades are per NCI CTC v3.0). ^4^ BM characteristics to be described: (i) BM MC burden (%) based on tryptase/CD117 (KIT) immunostaining, (ii) cytogenetics, and (iii) *KIT* D816V status. ^5^ Complete resolution of all evidence of organopathy unless observed changes are deemed related to treatment. ^6^ No progression in other elements of organopathy should be evident unless observed changes are deemed related to treatment. ^7^ Cytogenetic remission is not required; cytogenetic response, if any, to be documented as follows: CR disappearance of previously documented chromosomal abnormality without appearance of new ones, and PR at least 50% reduction of cytogenetic abnormality. ^8^ Per NCI CTC v3.0. ^9^ Given the difficulty in distinguishing treatment-related symptoms from disease-related symptoms.

**Table 3:** IWG-MRT-ECNM and Modified IWG-MRT-ECNM definitions of evaluable organ damage and response.

	IWG-MRT-ECNM Definition	IWG-MRT-ECNM Response Criteria	mIWG-MRT-ECNM Modifications
**Non-hematologic organ damage**			
Ascites or pleural effusions	Symptomatic ascites or pleural effusion requiring medical intervention such as: Use of diuretics (grade 2) *or* ≥2 therapeutic paracenteses or thoracenteses (grade 3) at least 28 days apart over 12 weeks before the start of treatment with one procedure performed 6 weeks before the start of treatment	Complete resolution of symptomatic ascites or pleural effusion (including trace/minimal on radiographic imaging) and no longer in need of diuretics for ≥12 weeks *and* No longer in need of diuretics for ≥12 weeks *or* No therapeutic paracenteses or thoracentesis for ≥12 weeks	Same as IWG-MRT-ECNM
Liver function abnormalities	≥Grade 2 abnormalities in direct bilirubin (>1.5 × ULN), AST (>3.0 × ULN), ALT (>3.0 × ULN), or ALP (>2.5 × ULN) in the presence of: Ascites *and/or* Clinically relevant portal hypertension, *and/or* Liver MC infiltration that is biopsy-proven *or* No other identified cause of abnormal liver function	Reversion of ≥1 LFTs to normal range for ≥12 weeks	Same as IWG-MRT-ECNM
Hypoalbuminemia	≥Grade 2 hypoalbuminemia (<3.0 g/dL)	Reversion of albumin to normal range for ≥12 weeks	Same as IWG-MRT-ECNM
Marked symptomatic splenomegaly	A spleen that is palpable >5 cm below the left costal margin and patient endorses symptoms of discomfort and/or early satiety	≥50% reduction in palpable splenomegaly (or ≥35% reduction in spleen volume based on 3D MRI or CT scan) and no endorsement of discomfort and/or early satiety for ≥12 weeks	**Definition:** Symptomatic or non- symptomatic splenomegaly palpable ≥5 cm below left costal margin. **Response criteria:** ≥35% reduction in spleen volume based on 3D MRI or CT scan for ≥12 weeks
Weight loss	N/A	N/A	**Definition:** Medically documented >10% weight loss in last 24 weeks ( ± 12 weeks) **Response criteria:** Reversion of >50% of weight loss in the 24 weeks preceding treatment
**Hematologic organ damage**			
Neutropenia	≥Grade 3 ANC (<1.0 × 10⁹/L)	≥100% increase *and* an absolute increase ≥0.5 × 10^9^/L for ≥12 weeks	Same as IWG-MRT-ECNM with allowance of CRh *
Anemia (transfusion-independent)	≥Grade 2 Hgb (<10 g/dL)	An increase in Hgb ≥2 g/dL that is maintained for ≥12 weeks	Same as IWG-MRT-ECNM with allowance of CRh *
Anemia (transfusion- dependent)	Transfusion of ≥6 units PRBCs in the 12 weeks before the start of treatment *and* Most recent transfusion occurring during the 4 weeks before the start of treatment *and* Transfusions administered for Hgb ≤8.5 g/dL *and* Reason for transfusions is not bleeding, hemolysis, or therapy-related	Transfusion independence for ≥12 weeks and maintenance of Hgb ≥8.5 g/dL at the end of the 12-week period of response duration	Same as IWG-MRT-ECNM with allowance of CRh *
Thrombocytopenia (transfusion- independent)	≥Grade 2 thrombocytopenia (<75 × 10⁹/L)	≥100% increase *and* an absolute increase ≥50 × 10^9^/L and no need for platelet transfusion for ≥12 weeks	Same as IWG-MRT-ECNM with allowance of CRh *
Thrombocytopenia (transfusion- dependent)	Transfusion of ≥6 units of apheresed platelets during 12 weeks preceding treatment *and* ≥2 units transfused during 4 weeks preceding treatment *and* Transfusions administered for platelet count <20 × 10⁹/L	Transfusion independence for ≥12 weeks *and* maintenance of platelet count ≥20 × 10^9^/L	Same as IWG-MRT-ECNM with allowance of CRh *

3D MRI, 3-dimensional magnetic resonance imaging; ALT, alanine aminotransferase; ANC, absolute neutrophil count; ALP, alkaline phosphatase; AST, aspartate aminotransferase; CT, computed tomography; Hgb, hemoglobin; IWG-MRT-ECNM, International Working Group-Myeloproliferative Neoplasms Research and Treatment; LFT, liver function test; MC, mast cell; MRI, magnetic resonance imaging; N/A, not applicable; PBRC, packed red blood cell; ULN, upper limit of normal. *CRh (CR with partial hematologic recovery) requires the following minimum levels for peripheral blood counts: absolute neutrophil count ≥0.5 × 10^9^/L with normal differential (absence of neoplastic mast cells and blasts < 1%) and platelet count ≥50 × 10^9^/L and hemoglobin ≥8.0 g/dL. Grade is based on the Common Terminology Criteria for Adverse Events, Version 4.03.

**Table 4:** Pure Pathologic Response (PPR) Criteria.

Response Category	Definition
Complete remission with full (CR) or partial (CR_h_) hematologic recovery ^a^	Bone marrow mast cell aggregates eliminated and serum tryptase <20 ng/mL
Molecular complete remission (molecular CR/molecular CR_h_)	*KIT* D816V mutant allele fraction falls below limit of detection by sensitive assay ^b^
Partial remission (PR)	≥50% reduction in bone marrow mast cells and serum tryptase level
Stable disease (SD)	Not in a CR, PR, or PD
Progressive disease (PD)	Transformation to acute myeloid leukemia (AML) or mast cell leukemia (MCL)

^a^ Partial hematologic recovery: ANC > 0.5 × 10^9^/L with normal differential (absence of neoplastic MCs and blasts < 1%) and platelet count >50 × 10^9^/L and Hgb level >8.0 g/dL. ^b^ *KIT* D816V allele-specific polymerase chain reaction or digital droplet assay with sensitivity ~0.1%. CR, complete remission; CR_h_, complete remission with partial hematologic recovery.

**Table 5:** A proposal for a protocol synopsis in advanced systemic mastocytosis with objectives reflecting tiered response criteria.

**Primary Objective** To determine the SM pathologic response rate (CR+PR) in patients with advSM
**Secondary Objectives** To determine the *KIT* D816V molecular response rate To determine the AHN pathologic response rate To determine the clinical response rate, measured by IWG clinical improvement (CI) To evaluate the EFS, PFS, LFS, and OS based on pre-treatment variables (advSM subtype; *S/A/R* status; prognostic score, e.g., IPSM, MARS, GPSM) and on-treatment variables such as SM pathologic response, molecular response, AHN pathologic response, and clinical response To evaluate time to initial SM pathologic response and duration of response To evaluate changes in the mutational profile with NGS compared to baseline and correlate with SM pathologic response, AHN pathologic response, EFS, PFS, LFS, and OS To evaluate changes in patient-reported symptoms using the AdvSM-SAF

SM: systemic mastocytosis; CR: complete remission; PR: partial remission; advSM: advanced systemic mastocytosis; AHN: associated hematologic neoplasm; IWG: International Working Group; EFS: event-free survival; PFS: progression-free survival; LFS: leukemia-free survival; OS: overall survival; *S/A/R*: *SRSF2/ASXL1/RUNX1*; IPSM: International Prognostic Score in Mastocytosis; MARS: Mutation-Adjusted Risk Score; GPSM: Global Prognostic Score in Mastocytosis; NGS: next generation sequencing; AdvSM-SAF: advanced systemic mastocytosis-symptom assessment form.

## Data Availability

Not applicable.

## Abbreviations

AdvSM	Advanced systemic mastocytosis
BM	Bone marrow
CR	Complete remission/response
CRh	Complete response with partial hematologic recovery
ECNM	European Competence Network on Mastocytosis
EMA	European Medicines Agency
FDA	Food and Drug Administration
HαT	Hereditary alpha-tryptasemia
ISM	Indolent systemic mastocytosis
IWG-MRT-ECN	International Working Group-Myeloproliferative Neoplasms Research and Treatment
MC	Mast cells
MCL	Mast cell leukemia
mIWG-MRT-ECNM	Modified International Working Group-Myeloproliferative Neoplasms Research and Treatment
MR	Major response
PPR	Pure pathologic response
PR	Partial response
SM	Systemic mastocytosis
SM-AHN	Systemic mastocytosis with an associated hematologic neoplasm
SSM	Smoldering systemic mastocytosis
WHO	World Health Organization
